# Simulated Heatwaves Affect Development of Two Congeneric Gregarious Larval–Pupal Endoparasitoids

**DOI:** 10.3390/insects17010025

**Published:** 2025-12-24

**Authors:** Lizhi Wang, Yanli Zhao, Zhihui Jiao, Baoping Li, Minghui Fei

**Affiliations:** State Key Laboratory of Agricultural and Forestry Biosecurity, College of Plant Protection, Nanjing Agricultural University, Nanjing 210095, China

**Keywords:** heat wave, *Oomyzus*, parasitoid, ladybird, climate change

## Abstract

Ongoing climate change is increasing the frequency and magnitude of high-temperature events, and extreme weather events such as heatwaves are expected to become more common. The impacts of extreme temperatures are likely even more severe at higher trophic levels, which depend on the adaptive capacity of lower trophic levels. However, compared with parasitoids at the third trophic level, less is known about these effects on parasitoids at the fourth trophic level, which occupy the terminal end of food chains. This study investigates the effects of experimental heatwave duration variability on the development of two congeneric gregarious larval–pupal endoparasitoids, *Oomyzus scaposus* and *O. spiraculus*, parasitizing the seven-spot ladybird, *Coccinella septempunctata* L. Heatwave treatments negatively affected the brood size of both species. The heatwave also affected the sex ratio of *O. scaposus*, though it remained highly female-biased. Exposure to heatwaves for 1 h accelerated the development of *O. scaposus*. For *O. scaposus*, the body mass of both females and males was lowest under the 3 h heatwave treatment. In contrast, for *O. spiraculus*, heatwaves had a marginally negative effect on the body mass of both female and male adults, though this effect was not statistically significant. Our results indicate that both species respond similarly, and even daily heatwave exposure of a few hours can affect the performance of *O. scaposus* and *O. spiraculus*.

## Simple Summary

Climate change is causing more frequent and intense heatwaves, which can disrupt food webs by affecting insects at the top of food chains. This study examined how short-term heatwaves influence two species of parasitoids that attack the larvae and pupae of seven-spot ladybirds, which are important predators of crop pests. We simulated heatwaves of different lengths and measured the performance of the two parasitoids. We found that even brief heatwaves reduced the brood size in both wasp species. For *Oomyzus scaposus,* short heat exposure sped up development, but longer exposure led to smaller adults. *O. spiraculus* showed similar patterns, but these were not statistically significant. Overall, the study shows that even short daily heat events can influence the performance of both species. Understanding these effects is important for predicting how climate change may alter predator–prey interactions, which could indirectly affect the natural control of crop pests and the stability of ecosystems.

## 1. Introduction

Climate change is altering thermal environments globally, resulting in an increase in the frequency and intensity of extreme temperature events [1], while also triggering ecological impacts across multiple scales [2,3]. During these events, daily maximum temperatures can surpass the upper thermal limits of local organisms. Even brief exposures of just a few hours to such high temperatures can be detrimental to ectotherms like insects, whose body temperature depends largely on environmental temperature [4,5,6].

The effect of extreme temperatures on insects can directly affect their physiology and behavior [7,8]. The responses of insect fitness to extreme temperature are likely to be both complex and variable, depending on insect life-history traits and the heat amplitude and heat frequency [9,10,11]. Although species can exhibit variable levels of thermal tolerance, exposure to extreme temperatures often results in a decrease in insect performance [5,12,13], such as growth and survival of immature stages [14], adult lifespan and reproductive success [15,16], and immunity and the accumulation of fat reserves [17].

Parasitoids are insects that deposit their eggs on or in a host organism (usually another insect or arthropod), which they use as a food source for their development, and eventually kill the host [18]. Parasitoid insects are vital top-down regulators of many insect herbivores, and there is a growing body of evidence that they have greater thermal sensitivity and lower thermal tolerance than their hosts [19,20]. Because parasitoids rely on complex physiological mechanisms to survive within their hosts, they are especially vulnerable to increasing temperatures, unpredictably variable temperatures, and stressful temperature events that could disrupt these processes [21]. The impact of extreme temperature is likely even more severe in parasitoids at higher trophic levels that depend on the capacity of those at lower trophic levels to adapt to these changes. However, compared with the parasitoids at the third trophic level, less is known about these effects on parasitoids at the fourth level at the terminal end of food chains [22]. Understanding the effects of climate variability on the fourth trophic level is of fundamental importance to understanding the ecological consequences of elevated temperatures.

The present study investigates the effects of experimental heatwave duration variability on the development of two congeneric gregarious larval–pupal endoparasitoids, *Oomyzus scaposus* Thomson and *O. spiraculus* Song, Fei & Cao (Hymenoptera, Eulophidae), of the seven-spot ladybird, *Coccinella septempunctata* L. (Coleoptera, Coccinellidae). Most ladybird beetles are attacked by primary parasitoids occupying the fourth trophic level [23], and these parasitoids have a potentially fundamental importance in mediating trophic cascades in natural communities. Several parasitoids of *C. septempunctata* have been previously described in the literature, but the biology, ecology, and developmental parameters of *O. scaposus* or *O. spiraculus* are thus far less studied [24]. Given that they are sister species and exhibit significantly overlapping traits, we hypothesize that both species respond to heatwave exposure similarly.

## 2. Materials and Methods

### 2.1. Insect Cultures

The prey for the seven-spot ladybirds, vetch aphids, *Megoura viciae* Mordvilko, originated from a long-standing laboratory colony maintained at the Jiangsu Academy of Agricultural Science. Seven-spot ladybirds, *C. septempunctata*, and their endoparasitoids, *O. scaposus* and *O. spiraculus*, were originally collected from vetch fields near Nanjing, east China. The ladybird colony was established from 30 adult seven-spot ladybirds randomly collected from the field and was maintained in insect rearing cages (40 × 40 × 40 cm) containing beans (*Vicia faba* L.) infested with vetch aphids as food resources. Colonies of *O. scaposus* and *O. spiraculus* were reared on host larvae. In total, 2 or 3 instar ladybird larvae were present at the end of a small brush applied to 2-to-3-day-old female wasps of each species in a plastic vial, and they were considered to be parasitized when the female wasp inserted her ovipositor into the host for at least 30 s. The parasitized larvae were then reared in a Petri dish (diameter of 5 cm) with vetch aphids that were replenished daily, and they were individually transferred into a glass tube after pupation. All insects were maintained in a climate room at a temperature of 22 ± 2 °C, a relative humidity of 50–70%, and a photoperiod of 16 L:8 D.

### 2.2. Experimental Design

Since in one of our previous thermal effects studies, we found that all parasitized seven-spot ladybirds (n = 33 for *O. scaposus,* n = 37 for *O. spiraculus*) had died at the pupal stage under an extremely high temperature of 33/28 °C; we dissected the pupae that have been found to be more than 90% parasitized but from which adult wasps could not successfully emerge. Therefore, we set a temperature of 33 °C to investigate the effect of variable heatwave duration on the performance of the two parasitoid wasps. We exposed the parasitized ladybird larvae to different heatwave (33 °C) durations using incubators (RGLC-P400-C3, Darth Cater, Hefei, China). The incubators were set with regular day–night temperatures of 22/20 °C, a relative humidity of 50–70%, and a photoperiod of 16:8 h L:D. In the middle of the daytime, the parasitoids were exposed to a 1 h or 3 h heatwave of 33 °C through their whole development period until adult wasps egressed. A group of parasitoid wasps that did not undergo any heat exposure served as the control.

A female wasp (3 days old) with a similar body size from each species was allowed to parasitize the host larvae once until leaving the host voluntarily. Each female wasp was used once. Each parasitized larva was transferred into a glass tube and put in incubators with a variable heatwave duration temperature with a relative humidity of 50–70%. The parasitized larvae were provided with vetch aphids until the adult wasps emerged. The development time from oviposition to adult eclosion, the number of emerging wasps (brood size), and the sex ratio (proportion of male adult parasitoids) were recorded. At eclosion, one male and five female wasps from each brood were dried in an oven at 60 °C for 72 h and weighed on a microbalance. The mortality rate (proportion of hosts that could not give rise to wasps) was also measured.

### 2.3. Statistical Analysis

Data were analyzed per parasitoid species and per sex if applicable (biomass). Data on mortality, brood sizes, sex ratio, adult body mass, and egg-to-adult development time were analyzed using general or generalized linear models depending on the distribution of the data. Mortality refers to individual trial failure (i.e., a host did not produce adult parasitoids). Brood sizes refer to the realized brood sizes, i.e., the number of wasps that successfully developed into adults. Adult body mass was the mean biomass of the parasitoids eclosed from the same host individual. All wasps from each host eclosed on the same day, resulting in a single data entry per host for development time. Sex ratio refers to the proportion of males per brood. Adult biomasses were log-transformed to meet assumptions of normality and homoscedasticity. In all statistical models, parasitoid species, temperature treatment, and their interactions served as explanatory variables. Mortality was analyzed using a GLM with a quasi-Binomial distribution and a logit link function. Brood size and development time were analyzed using a GLM with a quasi-Poisson distribution and a log link function to correct for overdispersion of the data. Sex ratio was analyzed using a quasi-Binomial distribution (correcting for overdispersion) and a logit link function. Post hoc Tukey HSD tests were performed to compare means when any of the model terms were significant. Statistics are provided when each of the main effect terms is entered last (Type III effects). All statistical analyses were undertaken with the R statistical software (version 4.4.3) [25].

## 3. Results

**Figure 1:**
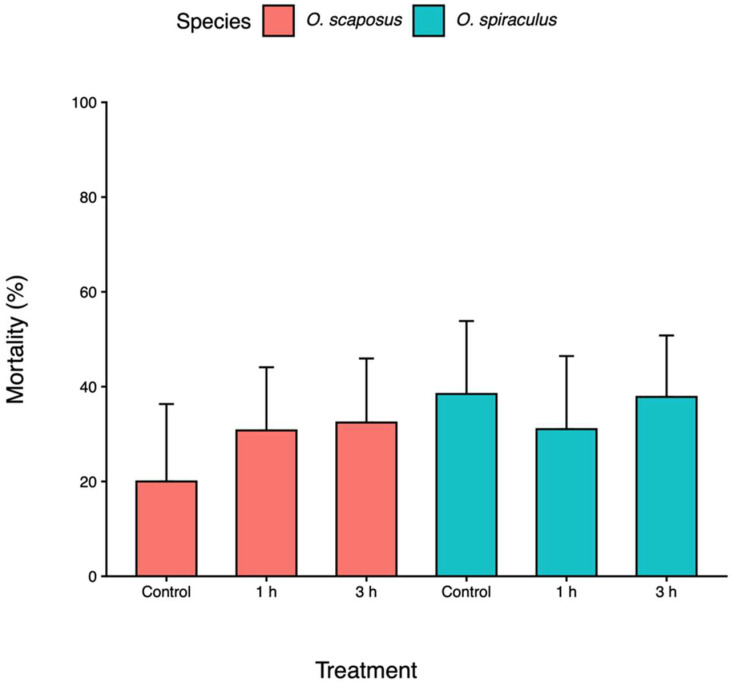
The mortality rates of *Oomyzus scaposus* (red) and *O. spiraculus* (green) under the control (no heat exposure) and different heatwave-duration treatments (1 h and 3 h at 33 °C). Bars represent the mean proportion of parasitized hosts that failed to produce wasps + SE.

Mortality. Mortality was neither affected by parasitoid species (F_1, 248_ = 0.43, *p* = 0.25), heatwave duration (F_2, 248_ = 0.43, *p* = 0.65), nor their interaction (F_2, 248_ = 0.34, *p* = 0.71). In general, mortality ranged between 20 and 37% (Figure 1).

**Figure 2:**
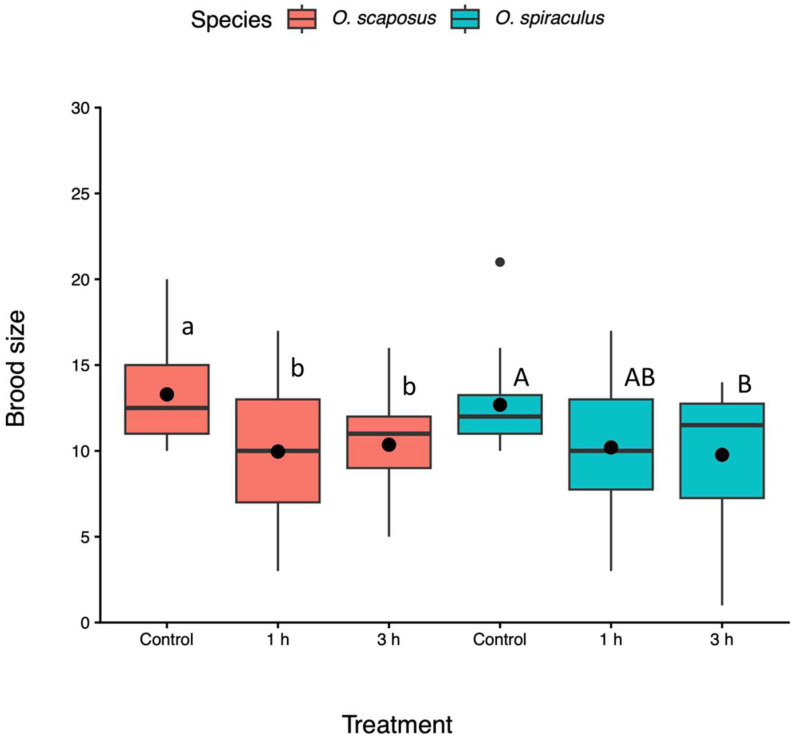
Brood size (number of emerging wasps) of *Oomyzus scaposus* (red) and *O. spiraculus* (green) under the control (no heat exposure) and different heatwave-duration treatments (1 h and 3 h at 33 °C). Data are presented as boxplots showing the interquartile range (median line inside) and mean values (black circles). Whiskers extend to 1.5 times the interquartile range, with points beyond considered outliers. Different letters above boxes denote significant differences between means within the same species across treatments (Tukey HSD tests, *p* < 0.05). Different small letters (a, b) denote significant differences for *O. scaposus*, while different capital letters (A, B) denote significant differences for *O. spiraculus*.

Brood sizes. The heatwave duration had a significant effect on parasitoid brood size (F_2, 167_ = 6.12, *p* < 0.01), and this effect was similar for the two parasitoid species (species: F_1, 155_ = 1.75, *p* = 0.18; heatwave–species interactions: F_2, 155_= 0.58, *p* = 0.56). The heatwave treatment negatively affected the brood size of *O. scaposus* (Tukey test: *p* < 0.05, Figure 2)*,* and there were no significant differences between the 1 and 3 h treatments (Tukey test: *p* > 0.05). *O. spiraculus* had a similar pattern; the brood size under the 3 h treatment was smaller than that under the control treatment (Tukey test: *p* < 0.05, Figure 2), and there were no significant differences between the 1 and 3 h treatments (Tukey test: *p* > 0.05).

**Figure 3:**
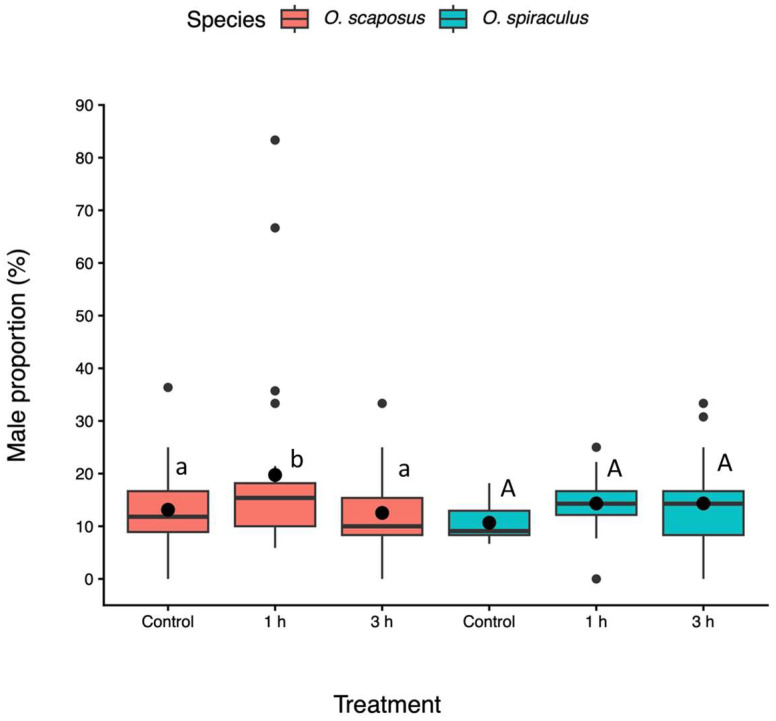
Proportion of males of *Oomyzus scaposus* (red) and *O. spiraculus* (green) under the control (no heat exposure) and different heatwave-duration treatments (1 h and 3 h at 33 °C). Data are presented in boxplots, which are explained in Figure 2. Different letters above boxes denote significant differences between means within the same species across treatments (Tukey HSD tests, *p* < 0.05). Different small letters (a, b) denote significant differences for *O. scaposus*, while the same capital letters (A) denote no significant differences for *O. spiraculus*.

Sex ratio. The sex ratio of both species was highly female-biased (Figure 3). The sex ratio of parasitoid offspring was affected by the heatwave treatment depending on the parasitoid species (heatwave: F_2, 21_ = 6.76, *p* < 0.01; species: F_1, 19_ = 0.13, *p* = 0.72; heatwave–species interactions: F_2, 20_ = 3.86, *p* = 0.023). For *O. scaposus*, the male proportion initially increased and then decreased as the heatwave duration increased (Tukey test: *p* < 0.05, Figure 3). The proportion of male *O. spiraculus* increased with heatwave duration, but this was not statistically significant (Tukey test: *p* > 0.05, Figure 3).

**Figure 4:**
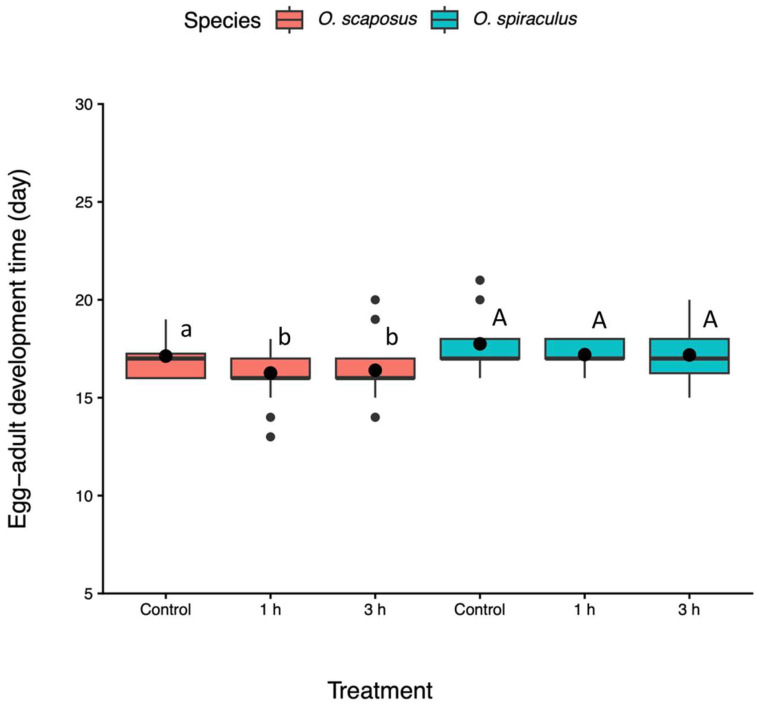
Development time (from oviposition to adult eclosion) of *Oomyzus scaposus* (red) and *O. spiraculus* (green) under the control (no heat exposure) and different heatwave-duration treatments (1 h and 3 h at 33 °C). Data are presented in boxplots, which are explained in Figure 2. Different letters above boxes denote significant differences between means within the same species across treatments (Tukey HSD tests, *p* < 0.05). Different small letters (a, b) denote significant differences for *O. scaposus*, while the same capital letters (A) denote no significant differences for *O. spiraculus*.

Development time. All males and females of the two parasitoid species eclosed on the same day from a single host. Egg-to-adult development time varied depending on the temperature treatment (F_2, 12_ = 3.70, *p* = 0.027) and parasitoid species (F_1, 12_ = 4.71, *p* = 0.032), but it was not affected by their interactions (F_2, 11_ = 0.067, *p* = 0.94). The development of *O. spiraculus* was longer than that of *O. scaposus* (Tukey test: *p* < 0.01, Figure 4). Heatwave exposure for 1 h could accelerate the development of *O. scaposus* (Tukey test: *p* < 0.05, Figure 4), and there were no significant differences between the 1 and 3 h treatments (Tukey test: *p* > 0.05). *O. spiraculus* had a similar pattern, but this was not statistically significant (Tukey test: *p* > 0.05, Figure 4).

**Figure 5:**
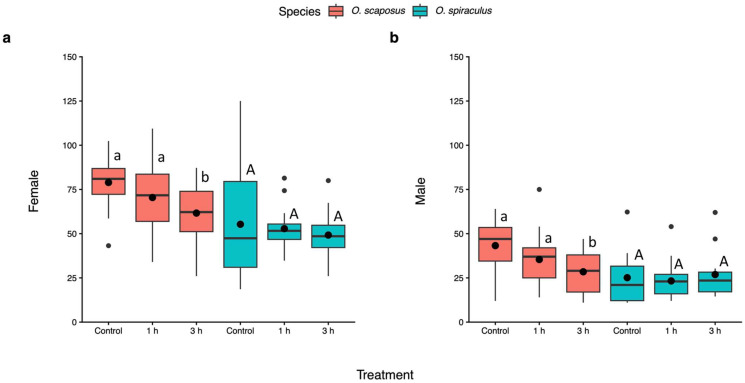
Adult body mass of *Oomyzus scaposus* (red) and *O. spiraculus* (green) under the control (no heat exposure) and different heatwave-duration treatments (1 h and 3 h at 33 °C). Panel (**a**) shows female mass, while panel (**b**) shows male mass. Data are presented in boxplots, which are explained in Figure 2. Different letters above boxes denote significant differences between means within the same species across treatments (Tukey HSD tests, *p* < 0.05). Small letters (a, b) denote differences for *O. scaposus*, and capital letters (A) denote no significant differences for *O. spiraculus*.

Adult body mass. Both female and male adult biomass were affected by the parasitoid species (female: F_1, 187_ = 22.40, *p* < 0.01; male: F_1, 183_ = 19.08, *p* = 0.01). The female adult body mass was also affected by the heatwave treatment (heatwave: F_2, 172_ = 7.03, *p* < 0.01; species–heatwave: F_2, 163_ = 1.70, *p* = 0.19). The heatwave treatment affected male adult body mass depending on the parasitoid species (heatwave: F_2, 176_ = 1.38, *p* = 0.26; parasitoid–heatwave interactions: F_2, 170_ = 4.67, *p* = 0.011). Both male and female wasps of *O. scaposus* were heavier than those of *O. spiraculus* (Tukey tests: *p* < 0.05, Figure 5). For *O. scaposus*, the body mass of both females and males was the lowest under the 3 h heatwave treatment (Tukey test: *p* < 0.05, Figure 5), whereas there were no significant differences between any other heatwave treatment comparisons. For *O. spiraculus*, the heatwave marginally negatively affected both female and male adult body mass, which was not statistically significant (Tukey test: *p* > 0.05, Figure 5).

## 4. Discussion

Our study provides clear evidence that transient, recurring heatwaves can significantly affect the performance of two congeneric endoparasitoids, *O. scaposus* and *O. spiraculus*. While mortality remained unaffected, the observed reductions in brood size for both species and changes in development time and adult body mass for *O. scaposus* suggest that heat stress can impose substantial sublethal fitness costs. These findings align with a growing body of literature demonstrating that the effects of extreme temperatures on insects are often more pronounced in sublethal performance traits than in direct survival [5,13].

The most consistent negative effect across both species was the reduction in brood size under heatwave conditions. This decline in the number of offspring reaching adulthood could be due to a number of underlying mechanisms, including a reduced egg hatch rate or increased mortality in developing larvae within the host under heat stress [6,14]. Heat stress reduces brood size, which directly influences population growth and stability. This is particularly critical for parasitoids at the fourth trophic level, like *Oomyzus* spp., which are already predicted to be more vulnerable to climate change due to their reliance on lower trophic levels and complex physiological interactions with their hosts [19,20]. Therefore, the heatwave effect on brood size, rather than mortality, may pose a greater threat to population persistence in the long term.

Local Mate Competition (LMC) theory is fundamental to understanding parasitoid ecology, predicting a female-biased sex ratio to minimize mating competition among male offspring—a pattern confirmed in both studied species. The highly female-biased sex ratio observed in both species is consistent with the expectations of LMC theory, which is common in gregarious parasitoids [26]. Even though the male proportion of *O. scaposus* significantly increased with the 1 h heatwave, it was still female-biased. One possible explanation for this result is differential thermal sensitivity between the sexes; however, the underlying mechanism requires further investigation. Furthermore, this observed increase in male proportion could contribute to a reduction in population growth potential, as fewer females are available for reproduction. Future studies should further investigate the ecological consequences of such sex ratio shifts under heat stress, including potential impacts on population dynamics. Insects are ectothermic animals, and their body temperature is easily influenced by the ambient environmental temperature. Previous studies have shown that high temperatures can accelerate the growth and development of insects [27,28]. In this study, we also found the accelerated development of *O. scaposus* even under a daily 1 h heatwave during its development. However, this accelerated development came at a cost, as evidenced by the significantly reduced adult body mass under the 3 h heatwave duration. *O. spiraculus* tended to have a similar pattern, but this was not statistically significant. This trade-off between development time and body size is a classic life-history constraint [29]. A smaller body size in parasitoids is often correlated with reduced fat reserves, shorter lifespan, and lower fecundity [30,31], which could undermine the apparent short-term benefits of faster development.

In conclusion, our results demonstrate that even short daily exposure to heatwaves can significantly impact the performance of two congeneric parasitoid wasps, the effects of which were more pronounced in sublethal performance traits than in direct survival. Given the crucial role of these parasitoids at the fourth trophic level in regulating ladybird populations and potentially mediating trophic cascades [24], a decline in their performance due to increasing heatwave events could disrupt the stability of this multi-trophic system. Consequently, future research should prioritize quantifying these sublethal effects across different trophic levels to better forecast the full ecological footprint of a warming climate.

## Data Availability

The original contributions presented in this study are included in the article. Further inquiries can be directed to the corresponding author.
